# Selective Scatterers Improve Efficiency and Color Neutrality of Semitransparent Photovoltaics

**DOI:** 10.1021/acsphotonics.5c02011

**Published:** 2025-11-06

**Authors:** Zheheng Song, Xi Lu, Oanh Vu, Jialu Song, Hiroshi Sugimoto, Minoru Fujii, Lars Berglund, Ilya Sychugov

**Affiliations:** † Department of Applied Physics, 7655 KTH Royal Institute of Technology, Stockholm 11419, Sweden; ‡ Department of Fibre and Polymer Technology, KTH Royal Institute of Technology, Stockholm 10044, Sweden; § Department of Electrical and Electronic Engineering, 12885 Kobe University, Kobe 657-8501, Japan

**Keywords:** Mie scatterer, color management, silicon nanoparticles, semitransparent photovoltaics, luminescent solar concentrator

## Abstract

We demonstrate that dielectric Mie scatterers, in the form of silicon nanoparticles (SiNPs), can enhance both the performance and esthetics of semitransparent photovoltaic devices. Unlike plasmonic metal counterparts, dielectric SiNPs exhibit lossless, narrow-band, spectral, and spatially tunable scattering in the visible spectral range. Their effect on a luminescent solar concentrator (LSC) with high visible light transparency is analyzed both theoretically and experimentally as a model system. By selectively reflecting a specific spectral band, SiNPs increase the optical path length of solar photons within the active layer, leading to improved absorption and hence device efficiency. Simultaneously, this light management strategy ensures transmitted color neutrality, an important requirement for wider acceptance of semitransparent photovoltaics. Numerical simulations show that in the regime of individual SiNPs with diameters around 160 nm, a submonolayer surface coverage of ∼10% is sufficient to achieve color neutrality, at the same time enhancing photocurrent by 10–15% for an LSC device. Experimentally, such a dispersed SiNP layer on an LSC substrate is realized by depositing NPs with the surface capped by a sacrificial polymer shell. Subsequent etching of the shell by oxygen plasma leads to an LSC device with a functional selective scattering layer in line with theoretical predictions.

## Graphical abstract

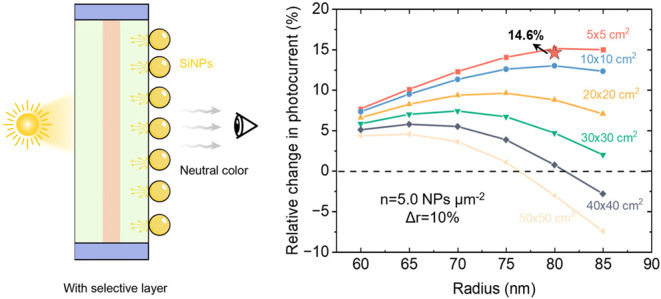

## 1 Introduction

As standard photovoltaics (PV) has advanced to maturity, different new and untapped previous directions are being explored now, such as semitransparent PV.^1–3^ It can be based on a variety of technologies. The most common approach is to reduce absorber thickness in a *p*–*n* junction to achieve a certain degree of visible light transparency.^4,5^ In this case, the fundamental property of semiconductors, namely, larger density of states at higher energy, often introduces a color to such thin films. For example, thin amorphous silicon (a-Si:H) devices are reddish, while thin-film direct bandgap semiconductors such as cadmium telluride (CdTe), copper indium gallium selenide (CIGS), or perovskite-based typically appear as red, yellow, brown, etc.^2^ Similar unwanted coloration effects are also relevant for some organic and dye-synthesized solar cell (DSSC) semitransparent PV devices, where the absorption is typically within a narrow spectral band in a visible range.^3^ Given that modern building standards favor neutral-colored glazing for most applications, with colored glass typically reserved for decorative purposes, achieving color neutrality in semitransparent PV is crucial for widespread application.^6^

One of the most straightforward remedy is external light management. A most common example would be Bragg gratings, which are thin films of exact composition (refractive index) and thickness designed to create a targeted spectral stopband.^7^ While those are common in small area optical filters, it might be difficult and costly to produce them on a large scale with required precision. Their performance is highly angle-dependent resulting in an excessively sharp, directional reflectance and performs poorly under diffused incident light. A more practical alternative would be selective scatterers based on nanoparticles (NPs). These can be deposited by a solution dispersion technique without any patterning or lithography. At the same time, efficient backscattering may increase solar light absorption by effectively increasing the optical path of sunlight through the device, thus acting as a retroreflector, such as *Tapetum lucidum* in some animal eyes.^8^ It should be noted that plasmonic metal NPs may not be the best candidates for this purpose. This is due to high intrinsic absorption and, hence, reduced scattering efficiency as well as limited spectral selectivity due to broadband responses.^9–11^ Luminescent solar concentrators (LSCs) have emerged as promising passive photonic devices. LSCs have attracted significant attention due to their scalable design, large sunlight absorption area, making them promising candidates for integration as “solar windows” in building facades.^12–15^ An LSC device absorbs sunlight, reemits it at a longer wavelength, and waveguides the light to the device edges, where it is collected by solar cells. Typically, LSC are composed of polymer host materials such as poly­(methyl methacrylate) (PMMA), with embedded fluorophores such as dyes or quantum dots (QDs).^16–18^ To enhance the stability of these devices, recent advancements introduced additional glass layers in a triplex geometry.^19,20^ These glass layers effectively reduce polymer degradation caused by environmental factors while minimizing light reabsorption in the polymer slab. With the advent of QDs as replacements for traditional dyes in LSCs, the performance of these devices has seen marked improvement in efficiency and photostability compared to organic dyes (rhodamine, etc.).^21,22^ Moreover, QDs are particularly favored due to their larger and controllable Stokes shift, which can reduce reabsorption during light propagation and improve the power conversion efficiency (PCE) of LSC devices.^18,19,23^

The use of QDs as absorbers, however, leads to the same type of coloration problems as for thin-film semiconductors due to similarity of their band structure and, hence, increasing absorption at shorter wavelengths. As a result, a yellowish or brownish tint (CIELAB color coordinate with b* > 10) often appears in LSC devices.^20,24^ The color in LSC can be adjusted through various methods, including the use of pigments and dyes as selective absorbers, structural colors from patterned microstructures, etc.^25,26^ However, many of these methods can reduce waveguiding of the luminescence and thus potentially degrade the optical efficiency of LSC. So, the requirements for application of such selective scatterers are even more stringent for LSCs than for thin-film *p*–*n* junction devices, as they should not simultaneously affect waveguided long-wavelength light.

To address this challenge, here we employed selective Mie-scattering nanoparticles (NPs), which have a scattering resonance at the wavelengths of interest (visible range), and much reduced scattering at the near-infrared (NIR) waveguiding range.^27^ Spherical silicon nanoparticles (SiNPs) offer several key advantages: their stable covalent-bond diamond-like structure ensures long-term photostability; their low fabrication cost and ease of application—whether by spraying or deposition onto a substrate—make them ideal for scalable, large-scale production.^28–30^ Furthermore, their selective scattering properties from ensembles of nearly monodispersed size particles can achieve color neutrality by scattering visible yellow light and maintaining low-loss waveguiding in the NIR region. As a result, enhanced PCE of LSC devices can be expected when the particle size and surface density are optimized.

Here, we first systematically investigated geometrical parameters of SiNPs, such as size, surface density, and interparticle distance, for this application by the finite-element method (FEM) and a Poisson distribution model. Our results revealed that NPs clustering substantially compromises selective scattering, reducing achievable color neutrality and efficiency gains. To mitigate formation of SiNP clusters in the experiment, we used poly­(*N*-isopropylacrylamide) (PNIPAM) polymer shells and plasma treatment, achieving a submonolayer (∼4–7 NPs/μm^2^) coverage with a large interparticle separation. Theoretical calculations were then validated by measuring the optical properties of such SiNP films. Finally, it was experimentally confirmed for LSC devices that, by leveraging Mie resonance, dispersed SiNPs effectively enhance both color neutrality and photocurrent. This strategy is broadly applicable to semitransparent photovoltaics, offering a versatile approach for optimizing light management in next-generation solar energy systems.

## 2 Results and Discussion

### 2.1 Finite-Element Method Simulation of SiNPs

First, we analyze the scattering behavior of an individual SiNP for this application. As particles are located on a glass substrate at the interface with air, an analytical Mie theory solution cannot be applied. Instead, we used the finite-element method to calculate scattering and absorption cross sections by solving the Helmholtz equation numerically. Based on the interaction of light with SiNPs with irradiation from the substrate side, we can define the following fractions: forward scattering (FS, to the air), backscattering (BS, to the substrate), absorption (ABS), and backscattering to the escape cone (EC, to the substrate within critical angle, ∼42°), as shown in Figure S1. The operational device geometry is that sunlight is first absorbed by the LSC, and therefore, SiNPs are placed on the backside (Figure 1a).

**Figure 1:**
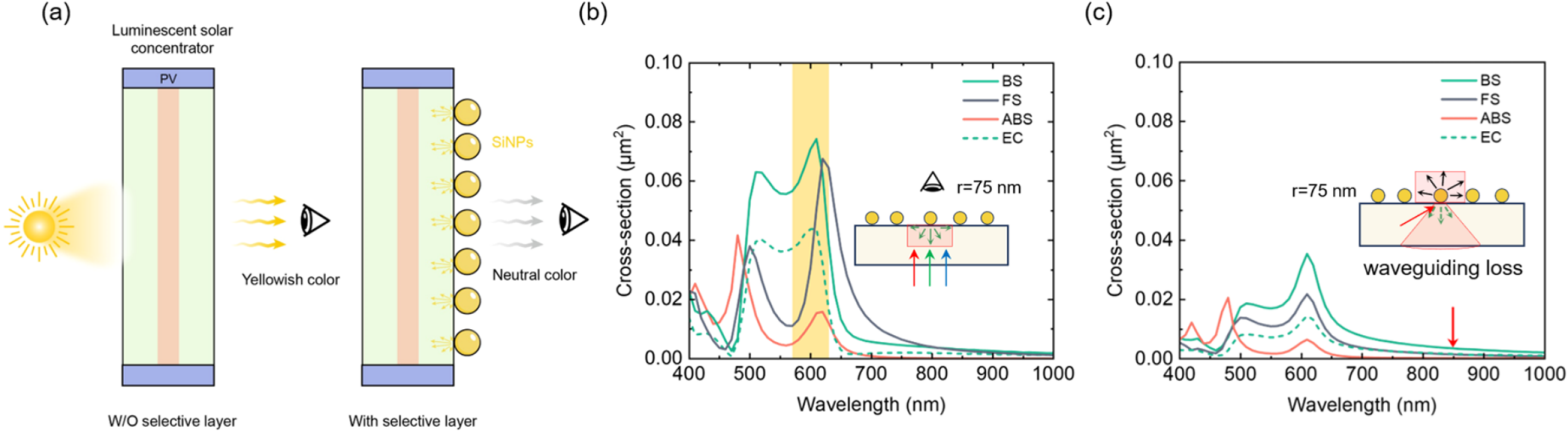
Scattering properties of SiNPs on LSCs. (a) Schematic illustration of LSCs with and without SiNPs, showing selective backward scattering and visual color neutrality. (b) Simulated spectral cross sections of backscattering (BS), forward scattering (FS), absorption (ABS), and escape-cone scattering (EC) at normal incidence (*r* = 75 nm). (c) Scattering behavior for waveguiding-mode light inside LSCs at 60° incidence, showing a SiNP-induced reduction of guided optical losses.

Solar light is initially considered at normal incidence, as shown in Figure 1b, and due to symmetry, only one polarization is treated. Functional SiNPs should have high BS in the yellow region (570–630 nm, yellow stripe in Figure 1b) and, at the same time, low ABS, EC, and FS to prevent light loss and haze across the entire visible spectrum in the LSC (Figure 1c). Additionally, as NIR photons from QD luminescence propagate within the LSC device, their total internal reflection (TIR) might be disrupted by the SiNPs. For our LSC device, we selected 850 nm as a characteristic QD luminescence peak wavelength and analyzed how the SiNP layer influences light in the waveguiding mode (red arrow in Figure 1c). As luminescence from spherical QDs is isotropic and unpolarized, we consider waveguiding modes impinging on the SiNP layer from the substrate at an average angle 60° from normal and calculate the average response for both incoming polarizations.

We observe that ABS, FS, EC, and BS probabilities are significantly weaker at 850 nm 60° tilted incidence compared with normal incidence in the yellow range. We initially selected the SiNP with a radius of 75 nm as the starting point for further optimization. At this NP size, the BS under normal incidence is found to be over 20 times stronger than its impact on NIR waveguiding losses, shown in Figure 1b,c. This selective scattering behavior is a key feature of the SiNPs utilized here, where strong resonant backscattering in the visible range coexists with very weak interaction for the NIR range. As also shown in Figure S2, we analyzed the size-dependent cross sections of SiNPs for these two cases in range of 55–95 nm. Under normal incidence, BS, FS, ABS, and EC all increase with the particle size, accompanied by a spectral red shift. A similar trend is observed for tilted incidence at 60° (the angle of waveguiding light with an average optical path). This set of data allowed us to identify the optimal NP size range for the purpose of this work.

So far, the response from individual SiNPs has been discussed. To represent a realistic situation of possible cluster formation, we also calculated the BS cross sections of clustered dimer SiNPs, as shown in Figure 2a, and both individual and clustered SiNPs follow similar trends. These effects are primarily attributed to near-field coupling at higher surface densities, where nanoparticles exhibit an increased probability of exciting unwanted optical loss channels—including leaky modes—that degrade performance. Larger particle radii result in higher BS, accompanied by a red shift and broadening of the reflectance peaks. For instance, individual SiNPs with a radius of 60 nm exhibit primary peaks between 430 and 530 nm. In contrast, for 90 nm SiNPs, the main backscattering peaks shift to 550–750 nm. Clustered SiNPs have their peaks red-shifted and reduced in amplitude. Particularly, the magnetic dipole peak (the right one) appears less pronounced compared to individual SiNPs. For instance, for SiNPs with an 80 nm radius at 600 nm, the BS cross section decreases from 0.066 to 0.048 μm^2^, a nearly 30% reduction. This trend indicates that the two primary peaks undergo broadening and amplitude reduction when SiNPs form clusters, which is an unwanted effect for this application. These results highlight the critical role of minimizing nanoparticle clustering to maintain spectral selectivity toward optimal optical performance.

**Figure 2:**
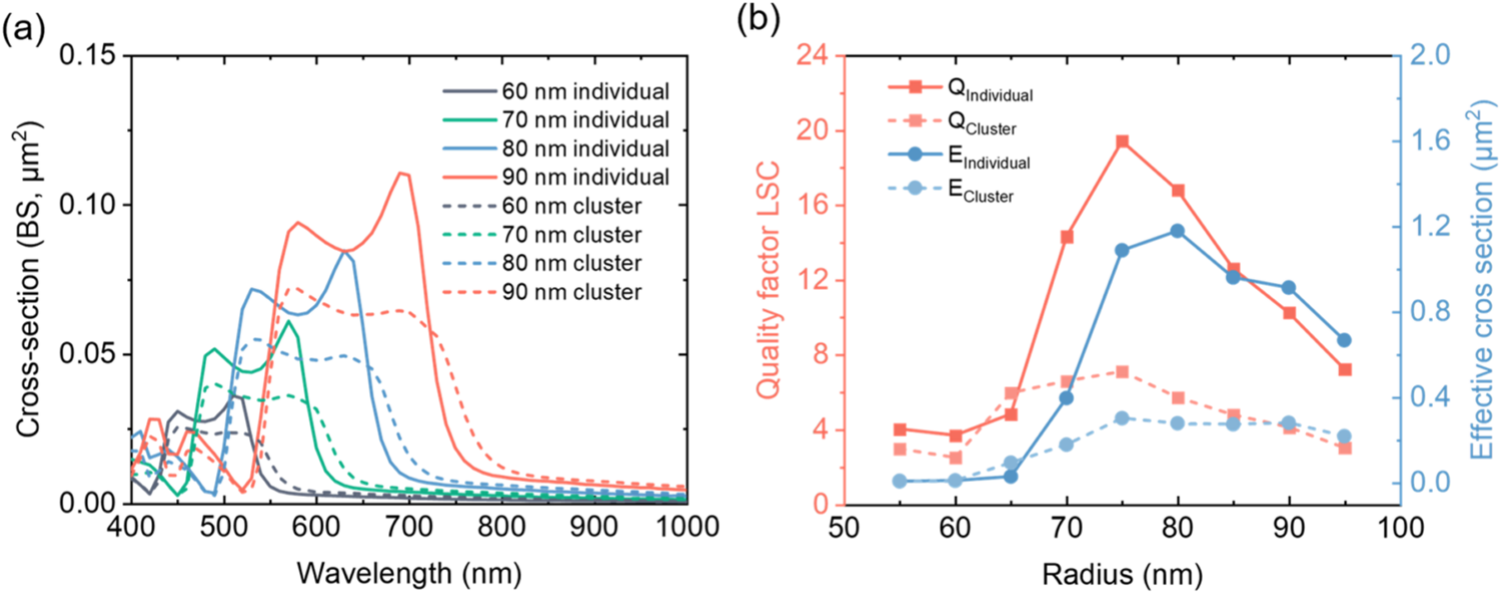
Scattering properties of individual and clustered SiNPs. (a) Simulated backscattering (BS) cross sections of individual and clustered SiNPs with radii ranging from 60 to 90 nm (only dimers are considered for simplification). (b) Calculated quality factor and effective scattering cross section quantifying the selective scattering performance of individual and clustered SiNPs.

In Figure S3a, we also analyzed the mutual influence between two SiNPs. As the spacing between them increases from 0 to 300 nm, the combined BS cross section gradually approaches the sum of two individual SiNPs, indicating a diminishing interaction. To quantify this effect, we plot the relative change in BS cross sections in Figure S3b. Setting a 20% variation as the threshold, we find that when the interparticle distance (surface-to-surface) exceeds 100 nm, SiNPs can be considered as nearly optically independent. This value will be used as a target for the experimental realization of separated SiNPs.

### 2.2 Modeling Distribution of SiNPs on a 2D Plane

To simulate macroscopic light–matter interaction properties of SiNP films, FEM faces limitations due to computational complexity of a large cell size. Therefore, an initial statistical estimation and simplification of the clustering process are necessary to guide experimental design and simulation analysis. So, we first estimate the clustering probability of SiNPs on a 2D plane, assuming purely random statistics. At low concentrations, SiNPs can be approximated as discrete points following a Poisson point sampling process. However, at a higher density, the finite size of SiNPs becomes nonnegligible, necessitating consideration of their diameter in the probability density function distribution. To account for the clustering process, we consider nearest-neighbor separation function in 2D.^31^ The probability density function (PDF) to find a nearest neighbor at a distance *r*

1 $$p_{1}\left(r\right)=2\pi \mathrm{Nr}\;\exp\;\left(-\pi Nr^{2}\right)$$

The probability density of particles with interparticle distance *p*(*r* < *D*) falling below the SiNP diameter *D* is then shifted to *p*(*r* = *D*), and they will be considered as dimers. As a first-order approximation, we leave out trimers and other higher-order clusters. As shown in Figure S4a, the 2D nearest-neighbor PDF function (blue curves) spans distances from 0 to 600 nm, for a ∼10% monolayer coverage of SiNPs with 75 nm radius. Since the SiNPs are first modeled as points, the resulting distribution is a Poisson distribution. The probability within a specific center-to-center distance range [*r*_1_,*r*_2_] can be obtained by integrating this PDF function

2 $$P_{1}\left(r\right)=\int_{r_{1}}^{r_{2}}2\pi \mathrm{Nr}\;\exp\;\left(-\pi Nr^{2}\right)\mathrm{dr}$$

Here, we take 20 nm as the bin size, and the red bar in Figure S4a depicts the probability of SiNPs with different spacing distances.

Notably, at this surface density for a random distribution, approximately 44% of such SiNPs already form clusters. This is an important result, indicating the need for preventive measures to avoid NP clustering on a glass surface even at surface density corresponding to ∼10–15% of a monolayer. We also quantify the percentage of SiNPs for different radii at the same surface density, as shown in Figure S4b, where larger SiNPs exhibit a higher tendency to cluster, as would be expected from purely statistical considerations.

### 2.3 Quantifying Selective Scattering and Color Neutrality from the SiNPs Layer

To quantitatively assess the selective scattering performance and potential light loss of the SiNP film on the LSC, we define two parameters, as given in eqs 3 and 4

3 $$Q_{\mathrm{LSC}}=\frac{\sigma_{\mathrm{yellow},\mathrm{BS}}}{\sigma_{850\;\mathrm{nm},\mathrm{FS}}+\sigma_{850\;\mathrm{nm},\mathrm{ABS}}+\sigma_{850\;\mathrm{nm},\mathrm{EC}}}$$

$$\sum_{\mathrm{eff}}=\sigma_{\mathrm{yellow},\mathrm{BS}}\cdot Q_{\mathrm{LSC}}$$ 4

Specifically, we examine the averaged BS of yellow light within the 570–630 nm range. The selective scattering quality factor *Q*_LSC_ is defined as the ratio of the probability of yellow sunlight being backscattered to the total probability of NIR waveguiding losses (FS, ABS and EC) induced by the SiNP layer. Beyond this ratio, the absolute value of the backscattered yellow light cross section is also important. Therefore, we introduce the effective BS cross section calculated as the product of the quality factor and the scattering cross section of the yellow band by SiNPs. These figures-of-merit comprehensively reflect the selective scattering performance, considering both efficiency and associated waveguiding losses.

We then plot the quality factor of selective scattering and its effective cross section to quantitatively examine their dependence on SiNPs radii. As shown in Figure 2b, for individual SiNPs, the quality factor reaches its peak at approximately *r* = 75 nm, with a maximum value of 20, while the highest effective BS cross section appears around 80 nm. In contrast, clustering significantly suppresses both the quality factor and the effective BS cross section, reducing the maximum quality factor to only 7. At the same time, its effective cross section drops from ∼1.2 to ∼0.4 μm^2^. Such a 3-fold reduction in both figure-of-merits due to clustering can substantially affect device performance, again highlighting the need for proactive clustering prevention.

### 2.4 Preparation of SiNP Films by the Langmuir–Blodgett Method

Size-selected polycrystalline SiNPs were fabricated by disproportionation of nonstoichiometric silica, as detailed elsewhere.^27^ Figure 3a shows the transmission electron microscopy (TEM) image of bare SiNPs (without polymer coating), with diameters ranging from 150 to 160 nm: the average diameter is ∼155 nm and size dispersion is ∼10%, which is in line with previous reports.^32^ This is the optimal size range for this application, as predicted from quality factor and effective cross-section calculations in Figure 2b. From the theoretical analysis above, it was established that a random distribution leads to a high clustering percentage, which compromises the optical performance of SiNPs. To achieve well-separated SiNPs deposition on the LSC device, we employed the Langmuir–Blodgett (LB) method. SiNPs with a PNIPAM outer layer (thickness ∼ 200 nm in solution) was dispersed in ethanol at a concentration of 250 μg/mL. The PNIPAM shell acts as a steric barrier, preventing direct contact and clustering of SiNPs.^33^ We note here that attempts to isolate NPs simply by surface charge were not successful, resulting in large cluster formation for any surface density > 1 NP/μm^2^. This was observed even for a large surface charge (+50 mV, through attachment of CTAB molecules on the SiNP surface) and for plasma-treated glass surfaces (with improved wettability).

**Figure 3:**
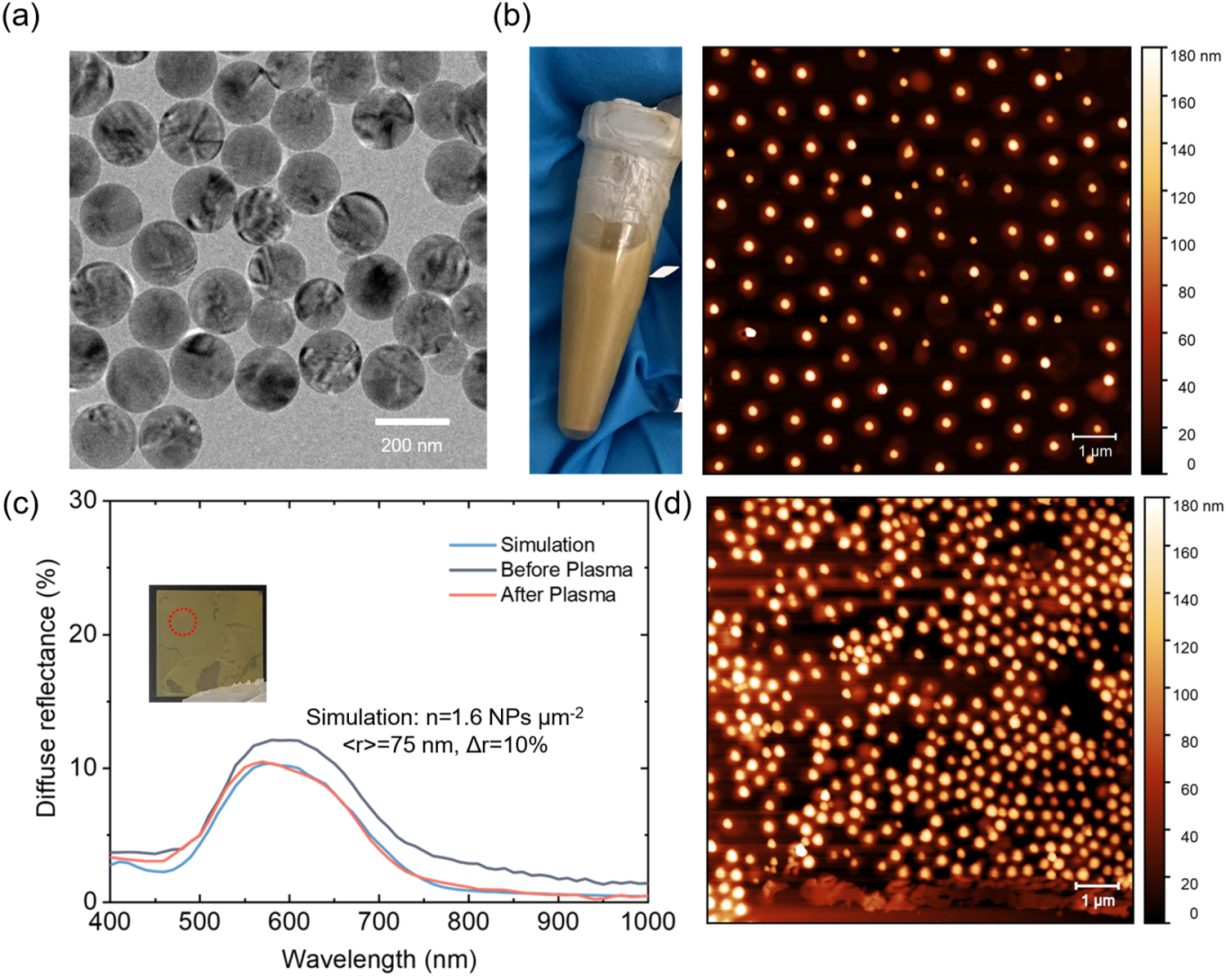
Preparation and characterization of SiNPs for LSC integration. (a) TEM image of as-synthesized SiNPs with diameters of approximately 150–160 nm. (b) Photograph of SiNP aqueous dispersion (left) and AFM image of a low-density SiNP monolayer (1.6 NPs μm^–2^) deposited on a glass substrate (right). (c) Diffuse reflectance spectra of SiNP films before and after plasma treatment, compared with simulation results at a low surface density; inset shows an optical photo of the film on a black background. (d) AFM image of a high-density SiNP film with a surface density of ∼5 NPs μm^–2^.

A 2.2 × 2.2 cm^2^ glass substrate was sequentially cleaned in acetone, ethanol, and deionized water using an ultrasonic bath for 10 min each. One side of the substrate was then covered, and the sample was exposed to air plasma treatment for 15 min, rendering only the exposed side hydrophilic. The NP solution was then spread on the surface of deionized (DI) water to form a film, which was subsequently transferred onto a glass cover, yielding a submonolayer. To achieve different density distributions of SiNPs, both the concentration and amount of SiNPs and beaker diameter were controlled. For example, to achieve a surface density of ∼4–7 NPs μm^–2^, a beaker with a diameter of 2.6 cm was filled with DI water. A premixed solution containing 200 μL of SiNPs (330 μg/mL in water) was prepared in advance. Then, the solution was centrifuged to remove the water solvent. Before the LB film preparation, 200 μL of ethanol was added to the vial to gently redisperse NPs. The final mixture was then carefully dispensed onto the water surface. Finally, the glass cover was used to lift the assembled layer for drying.

To remove the PNIPAM layer and minimize its influence on optical properties, air plasma treatment was applied at 20 W for 30 min on thus prepared SiNPs on glass. This process ensured a sharper refractive index contrast between the SiNPs and their surroundings, bringing the film closer to the simulation conditions. We prepared both low and high surface density samples. The low-density film exhibits better uniformity across the surface, with the local surface coverage ranging from 1.4 to 1.8 NPs μm^–2^. In contrast, higher-density samples—with a target surface coverage of ∼5 NPs μm^–2^—often exhibit larger spatial variations, ranging from 3.5 to 7 NPs μm^–2^. Furthermore, at a low density, interparticle interactions are minimal already from statistical considerations, allowing for a clearer comparison with theoretical models based on scattering from independent NPs. At a higher density, however, multiple scattering and near-field couplings may introduce deviations. Additionally, since atomic force microscopy (AFM) imaging tends to broaden particle features by tip convolution, low-density films are preferred for evaluating morphological properties. Higher-density films are, on the other hand, more pertinent for device operation.

Figure 3b displays the yellowish appearance of SiNPs@PNIPAM dispersed in water along with the corresponding AFM image of the low-density film on a glass substrate without clusters. For the higher-density film in Figure 3d, we could also confirm good interparticle separation, largely thanks to the PNIPAM shell steric effect. The experimentally obtained nearest-neighbor distance distribution reveals an average interparticle distance of ∼350 nm in Figure S5a—substantially higher than the 100 nm threshold used to define individual single-particle conditions. This again confirms the lack of clustering effects even in a higher density.

To quantify the plasma treatment effect, we analyzed more than nine images from approximately the same region on the sample (before and after plasma treatment), covering approximately 1200 NPs in total, focusing on the NP height. The localization method used to track the AFM measurement area after plasma treatment is shown in Figure S5b. The resulting size histogram, fitted with a Gaussian curve, reveals that the peak SiNP height shifts by ∼10 nm (from 155 to 147 nm) after plasma treatment. The effect of plasma treatment was also observed by color change of such films in the reflection from yellow to green (Figure S6). From previous cross-sectional scanning electron microscopy data,^27^ it was revealed that the PNIPAM polymer shell largely collapses after solvent deposition and drying up while still preserving its function as a steric hindrance. This explains a relatively small change in the NP height observed here with AFM when the polymer shell is etched away. Altogether, these results confirm that plasma treatment effectively removes the PNIPAM shell, although it only slightly reduces the observable height of SiNPs. It ensures cleaner, separated NPs close to the individual NP approximation regime, which will be used for subsequent optical analysis. As shown in Figure S7, the excellent durability of Mie-scattering SiNPs is confirmed by the 500-h aging test (simulating over 1 year of outdoor exposure), which resulted in only a minor spectral change. The slight variation in reflectance is attributed to a minor (a few millimeters) shift in the measurement position. As far as mechanical stability is concerned, one can point out that the NP film will be placed facing inward in an insulating glazing unit, i.e., toward the space between glass panes. Then, the NP layer is exposed to only air or inert gases, such as argon, used for thermal insulation, which is beyond the disruptive mechanical reach of the elements or humans.

### 2.5 Optical Characterization of SiNP Films

To validate our simulations and analysis, first, we calculated the film reflectance values based on the cross-sectional results from the FEM method and given surface density, assuming a Gaussian size distribution for the particles. For a given SiNP radius, we incorporate a size distribution based on the Gaussian PDF, with a 10% core radius variance as measured from TEM (Figure 3a). It should be noted that the AFM data presented in Figures 3b,d and S6 were acquired prior to plasma etching and may include contributions from the polymer shell or its residuals. As such, the particle size measurements may be less accurate, and these images are used solely for estimating surface density and not for precise size analysis. By integrating the PDF, we determined the fraction of particles within different size ranges (20 nm bin size). For instance, for SiNPs with a nominal radius of 80 nm, the distribution includes 25% at 80 nm, 21% at 75 and 85 nm, and 11% at 70 and 90 nm. The final reflectance is obtained by weighing each radius contribution accordingly and determined as the product of NPs cross sections and surface density: *R*(λ) = σ­(λ)*·N*. Larger particle radii lead to increased reflectance, accompanied by a red shift and broadening of the reflectance peaks. Note that the FEM simulation assumes no diffuse reflectance from the glass substrate under normal incidence. However, integrating sphere measurements (Figure S8) reveal ∼2–3% diffuse reflectance in the infrared region for a pure glass reference sample, which was subtracted to obtain the NPs-only signal for comparison with simulations.

As shown in Figure 3c, before plasma treatment, the measured reflectance spectrum (for ∼1.6 NPs/μm^2^ sample with ∼150–160 nm diameter corresponding to the low-density sample in Figure 3b) exhibits a broad peak centered around 600 nm, deviating from the simulated data most likely due to the polymer shell’s influence. After plasma treatment, the reflectance curve reduced in intensity and started to follow more closely the simulated trend *R*(λ). This again confirms that plasma treatment modifies the SiNPs surface, enhancing agreement with theoretical predictions corresponding to the individual NP regime. These results demonstrate a good match between our simulations and experimental measurements for the films of SiNPs of a specific radius, validating the calculated selective scattering behavior.

### 2.6 LSC Device with an SiNP Layer

To experimentally verify the concept at the device level, we fabricated a SiNPs integrated luminescent solar concentrator (LSC@SiNPs) device. A 5 × 5 cm^2^ LSC was made in a triplex glass geometry with a polymer layer doped with metal nanoclusters.^200^ Another thin glass substrate was coated with SiNPs, followed by the application of a thin layer of refractive index-matching oil on the other side. The SiNP-coated substrate was then attached to the LSC device, ensuring the removal of air bubbles to minimize optical distortion. The final surface density was estimated to be approximately 5 NPs μm^–2^, corresponding to 10–15% monolayer coverage, which was selected to achieve color neutrality for integration with LSC devices (cf. higher concentration film AFM image in Figure 3d).

As shown in Figure 4a, we measured the total reflectance under normal incidence, which includes both specular and diffuse components for the SiNP film. A significant enhancement in reflectance was observed in the 500–700 nm range—corresponding to the yellow-red region—while reflectance in the NIR range remained low. Compared to the previously studied low-density samples, the high surface density induces stronger interparticle interaction and prominent near-field coupling, leading to larger deviation from theoretical predictions. Deviation from theoretical predictions was observed. Nonetheless, the main predicted function of a high-density SiNP layer—specifically, its influence on the reflectance peak position and the reflectance intensity—was unequivocally confirmed by the experimental data, eliminating the need for any fitting parameters.

**Figure 4:**
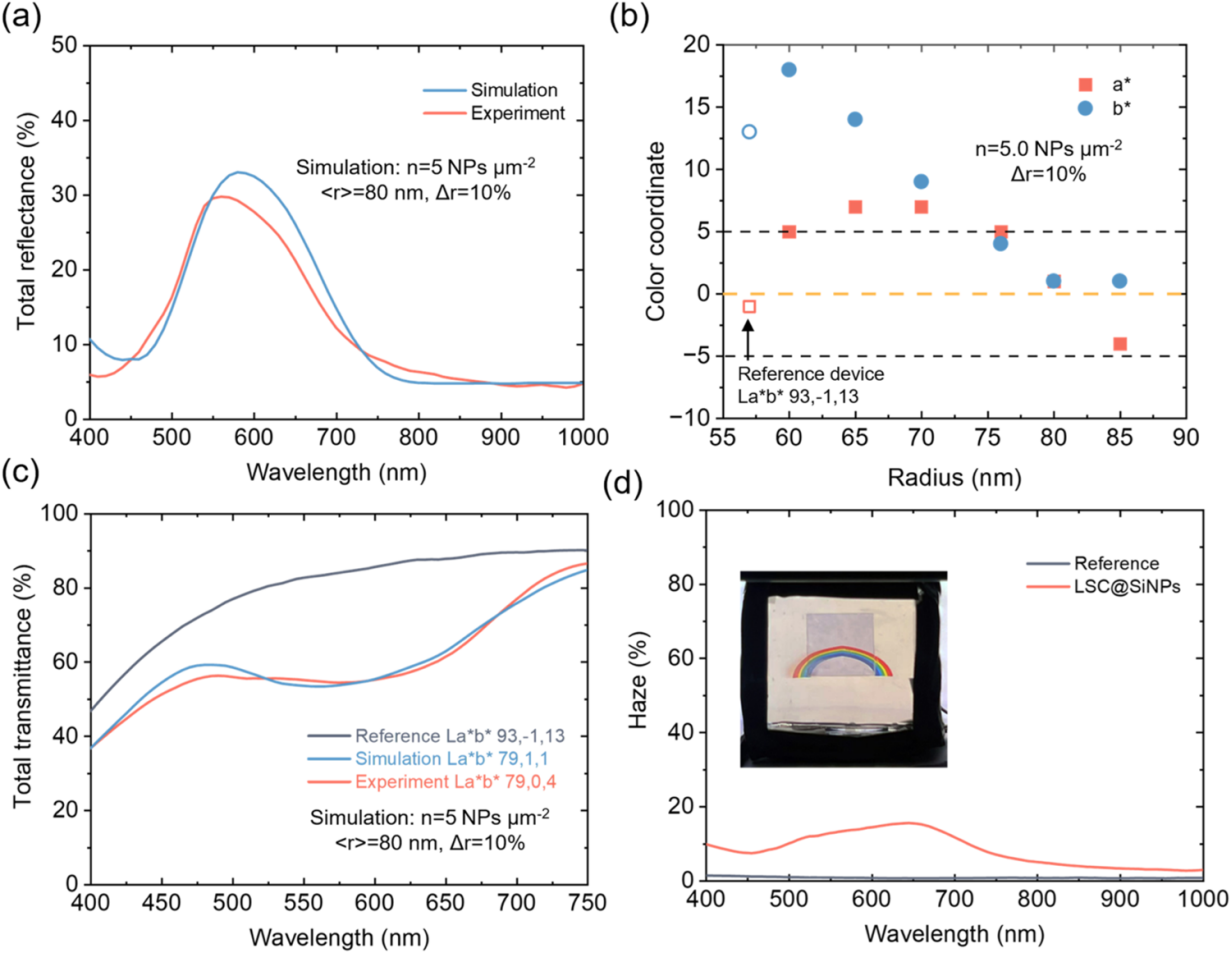
Optical characterization of LSC@SiNPs with high surface coverage. (a) Comparison of simulated and experimental total reflectance spectra for SiNP films (*n* = 5 NPs μm^–2^, ⟨*r*⟩ = 80 nm, Δ*r* = 10%), weighted by a Gaussian size distribution. (b) Simulated CIELAB color coordinates (a*, b*) of LSCs with different SiNP sizes, compared with the reference device (Lab = 93, −1, 13). (c) Total transmittance spectra of the reference and LSC@SiNPs (*r* = 80 nm), from both simulation and experiment. (d) Haze spectra of the reference and LSC@SiNPs; inset shows visual appearance under a solar simulator with a white reflector.

To estimate expected color neutrality, we investigated how SiNPs of different radii influence the appearance of the LSC. The transmittance of LSC devices with incorporated SiNPs was calculated based on individual particle absorption and backscattering cross sections using the following expression

5 $$T\left(\lambda \right)={1-\sigma }_{\mathrm{ABS}}\left(\lambda \right)\cdot N-\sigma_{\mathrm{BS}}\left(\lambda \right)\cdot N$$

where *N* is the surface density of SiNPs. To quantify the color appearance, we applied the CIELAB color space. The *L*, a*, and b* values were derived from the transmittance spectra in the visible wavelength range (400–750 nm).^34^ As shown in Figure 4b, the theoretical results indicate that a SiNP radius of 80 nm yields a nearly color-neutral appearance for such a device. Based on this finding, we further investigated the influence of surface density on the color coordinates, as shown in Figure S9a,b. Notably, a radius of 80 nm and a surface density of ∼5 NPs/μm^2^ correspond to optimal performance in this respect.

In the experiment results in Figure 4c, the reference LSC device exhibits a monotonic increase in transmittance from 45% at 400 nm to 90% at 750 nm due to metal nanocluster absorption. Upon integrating an SiNP layer, a pronounced transmittance drop occurs in the 500–700 nm region. This reduction in transmittance helps to suppress spectral color contrast, thereby rendering the device more color-neutral in appearance. We compared the experimentally measured and simulated transmittance spectra of the LSC@SiNPs system. Both curves display a similar monotonic increase in transmittance from ∼40% at 400 nm to over 80% at 750 nm, with a steeper rise beyond 600 nm. While the overall trend is consistent, some minor fluctuations are observed in the simulated spectra between 450 and 550 nm, while generally the experimental results align well with the model predictions. In addition, we compared the CIELAB color coordinates. Notably, the a* value remains relatively unchanged, whereas the b* component—associated with yellow-blue tones—shows a substantial reduction, which is desirable for improved color neutrality. The measured values were (79, 0, 4), while the simulation predicted (79, 1, 1), demonstrating good agreement. This confirms that the LSC@SiNPs system achieves near color-neutral transmission when compared to the reference LSC device of a yellowish tint (93, −1, 13). The strong agreement between experimental and theoretical results confirms that selective scattering from SiNPs can be effectively tuned to enhance the visual and optical performance of semitransparent photovoltaic devices.

We also report the haze of the LSC@SiNPs device in Figure 4d. The bare LSC exhibits a low haze level of approximately 2% across the entire visible range. Upon integration with SiNPs, a moderate increase in haze is observed, particularly in the 500–700 nm range (yellow-red region), which is attributed to Mie-resonant forward scattering. Nevertheless, the overall haze remains relatively low, especially in the spectral range of highest eye sensitivity (∼10% at 500 nm), indicating minimal impact on glazing. A photograph of the device with a 1-sun solar simulator background on white paper (printed with rainbow) (inset of Figure 4d) visually demonstrates the low haze and the color neutrality effect. The reference LSC area shows a distinct yellowish hue in transmission mode, whereas the LSC integrated with SiNPs appears more color-neutral with a greyish tone. This provides strong experimental validation that SiNPs can effectively tailor the optical properties of LSCs, both quantitatively and visually.

### 2.7 Photovoltaic Performance of the LSC Device with SiNPs

Now we are in a position to examine the impact of SiNPs on LSC photovoltaic efficiency. Introducing selective scatterers on the backside of an LSC device influences its performance in multiple ways (as shown in Figure S10 and Supporting Information Note 2). First, the backscattered light that enters the escape cone can exit the device after partial absorption by the LSC fluorophores, thereby extending the optical path length and enhancing the solar light absorption within the LSC. This absorption enhancement is wavelength-dependent. Second, backscattered light that remains outside the escape cone undergoes waveguiding through TIR. This light may either be absorbed by fluorophores or guided to the LSC edges for solar cell harvesting. Alternatively, it can be scattered out by the SiNPs layer at the following bounce events. While the former two mechanisms contribute to photocurrent enhancement of LSC devices, the SiNPs layer also introduces an additional loss channel for waveguided photons generated by the LSC fluorophores, such as FS, ABS, and EC cone losses. In general, the photocurrent *I*_0_ of an LSC is proportional to the absorbed solar power fraction *A*_0_ and the waveguiding efficiency η_WG_.^20,35,36^ When incorporating SiNPs, two additional factors modify the absorbed solar fraction, and the waveguiding efficiency is adjusted accordingly

$$I_{\mathrm{ss}}∼\left(A_{0}+A_{\mathrm{EC}}+A_{\mathrm{WG}}\right)\cdot \eta_{WG_{\mathrm{SS}}}$$

Here, *A*_EC_ represents the absorbed fraction of light that is backscattered by the SiNPs and remains within the EC. *A*_WG_ refers to the absorbed fraction of BS light that enters the waveguiding mode. Finally, η_WG_SS__ denotes the newly reduced waveguiding efficiency due to the presence of SiNPs.

The waveguiding efficiency depends strongly on the device area. Here, we analyzed square LSCs of different side lengths: 5, 10, 20, 30, 40, and 50 cm. As the device area increases, the average number of photons bounced before collection by solar cells also grows. As a result of these calculations, we can see that the photocurrent enhancement reaches 5–20% at an optimized SiNPs radius of around 60–80 nm, which also corresponds to the optimal conditions for achieving good color neutrality. As shown in Figure 5a, for individual SiNPs, the photocurrent increases with the particle size, peaking at 80 nm before slightly decreasing. This enhancement is most pronounced for smaller device areas (5 × 5, 10 × 10, and 20 × 20 cm^2^), while for larger devices (30 × 30, 40 × 40, and 50 × 50 cm^2^), the effect diminishes, indicating that the scattering-induced losses start dominating at larger scales. Regarding relevant values, Figure 5a shows that even for areas up to >50 × 50 cm^2^, a net improvement in photocurrent is theoretically still possible with optimized SiNP radii (e.g., 70 or 75 nm for a +5% increase). So, if the photocurrent increase is preferred instead of a complete color neutralization, a different NP size can be chosen.

**Figure 5:**
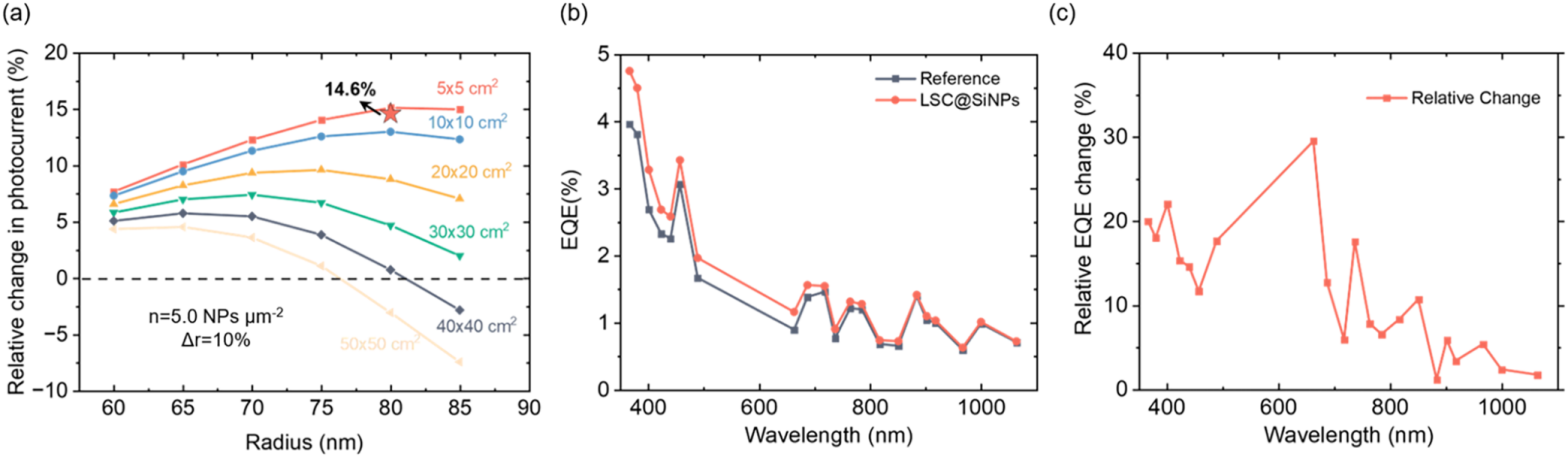
Photovoltaic performance enhancement of LSC@SiNPs. (a) Simulated relative photocurrent enhancement as a function of SiNP radius and LSC size; the star symbol indicates the experimental result (14.6%) for a 5 × 5 cm^2^ device. (b) EQE spectra of the reference and LSC@SiNPs devices under AM1.5G illumination. (c) Relative change in EQE extracted from panel (b) as a function of the wavelength.

Alternatively, clustered SiNPs exhibit a notably weaker enhancement, as seen in Figure S11. The photocurrent increase is lower across all device sizes, with minimal or even purely negative changes for larger areas. Here, we note that the losses relevant to LSC due to PL photon waveguiding disruption are nonexistent for other semitransparent PV technologies, such as organic PV or thin-film *p*–*n* junctions. So, the device size limitations discussed here will not be applicable beyond the LSC field, and the net photocurrent enhancement can be expected due to optical path increase through the active layer in them.

Then, we used a 5 × 5 cm^2^ LSC device with solar cells all around its perimeter to experimentally demonstrate proof-of-concept. Here, the backside of the LSC was fully covered with SiNPs in a high-density mode (∼5 NPs/μm^2^). The star symbols in Figure 5a represent the measured photocurrent increase under 1-sun illumination using a solar simulator. Compared to the reference device, the LSC@SiNPs show a 14.6% increase in photocurrent, with the short-circuit current rising from 3.16 to 3.60 mA. The corresponding current–voltage (*I–V*) curves are provided in Figure S12. As shown in Figure 5b, we also measured the external quantum efficiency (EQE) of both the reference and LSC@SiNPs devices to unravel spectral contributions to this increase. Fluctuations in the EQE data, especially at longer wavelengths, originate from systematic calibration and uniformity variations of the solar simulator. To illustrate device performance differences more clearly, Figure 5c presents the corresponding relative EQE enhancement. A pronounced increase is observed in the 500–700 nm range, which aligns well with the designed selective scattering region of the SiNPs. This enhancement is consistent with our photocurrent predictions and further validates the role of SiNPs in improving the spectral harvesting efficiency.

## 3 Conclusions

In summary, we propose a method using Mie-scattering SiNPs to achieve color neutrality and enhance the efficiency of semitransparent PV LSC devices. Through simulations, we analyzed both individual and clustered SiNPs configurations, confirming that clustering degrades the selective scattering performance. Experimentally, we fabricated SiNPs@PNIPAM submonolayer films using the LB method and subsequently removed the polymer shell via plasma treatment to ensure the SiNPs remained well-dispersed. Our results firmly confirm that integrating SiNPs with LSCs enables highly effective selective scattering, with reflectance and transmittance measurements closely matching the simulations. Additionally, we conducted an analytical analysis of the influence of SiNPs on the LSC photocurrent, revealing a positive and significant effect for optimal sizes and validating this experimentally, contributing to the overall efficiency enhancement. This approach provides a versatile and robust strategy for external light management and can be extended to other devices, such as organic or thin-film solar cells or other semitransparent PV systems requiring external light management.

## 4 Experimental Section

Electromagnetic simulations were performed using COMSOL Multiphysics 6.2 (Wave Optics module) by solving the Helmholtz equation numerically. The simulation first calculates the electric field distribution in the absence of silicon nanoparticles (SiNPs). Next, the scattering properties of the SiNPs are analyzed by using a perfectly matched layer (PML) boundary condition to minimize artificial reflections. Detailed definitions and simulation parameters are provided in Figure S1. SiNPs were synthesized via disproportionation of nonstoichiometric silica from silicon monoxide, as previously reported.^27^ They were coated with PNIPAM by polymerizing this microgel shell on 3-(trimethoxysilyl)-propyl methacrylate functionalized SiNPs via radical polymerization. They were deposited on plasma-treated low-iron glass substrates by using the Langmuir–Blodgett method. For this, SiNPs were first centrifuged to remove the excess solvent and then redispersed in ethanol in ∼1:1 volume ratio. A small volume of the suspension was carefully pipetted onto the air–water interface of deionized (DI) water. Once a uniform submonolayer formed at the interface, a glass substrate was vertically dipped into the region, exhibiting optimal nanoparticle coverage to transfer the monolayer. PNIPAM shells were removed by air plasma treatment (30 min, 91,000-PELCO easiGlow). Optical oil with a refractive index matched to that of the LSC device glass was used to eliminate air bubbles between the SiNP film glass and the LSC substrate—a step to minimize optical losses caused by refractive index mismatch. The surface morphology was characterized by using atomic force microscopy (JPK NanoWizard 3, contact mode) and transmission electron microscopy (JEM-2100F, JEOL, 200 kV). Photon budget performance was evaluated using a laser-driven xenon plasma light source (Energetiq EQ-99) coupled with a monochromator (SP2150i), a 6 in. integrating sphere (Labsphere), and a Peltier-cooled CCD spectrometer (Princeton Instruments, −75 °C). Photovoltaic measurements were conducted under 1-sun (AM1.5G) illumination using a class AAA+ solar simulator (G2 V Sunbrick) and a Keithley 2450 source meter, with nonactive areas masked using a black tape. The SiNP films dispersed in ethanol solution were also deposited using a 100 mm head Ossila Slot-Die Coater with a dispensing rate of 10 mm/s, a substrate temperature of 50 °C, and a solution concentration of approximately 4 mg/mL. The slot-die deposition was performed with a deposition gap of ∼400 μm, a substrate temperature of 45 °C, and a deposition speed of 5 mm/s. The ∼3.0 NPs μm^–2^ film was prepared using 10 deposition cycles, and the ∼7.0 NPs μm^–2^ film was achieved using 30 deposition cycles. The substrate is 2-mm-thick glass substrate cut in the size of 12 × 5 mm^2^. The SiNP concentration in the slot-die ink was ∼4 mg/mL. The slot-die deposition was performed with a deposition gap of ∼400 μm, a substrate temperature of 45 °C, and a deposition speed of 5 mm/s. The ∼3.0 NPs μm^–2^ film was prepared using 10 deposition cycles, and the ∼7.0 NPs μm^–2^ film was achieved using 30 deposition cycles. The substrate is a 2-mm-thick glass substrate cut in the size of 12 × 5 mm^2^. Accelerated aging test was performed in a UV climate chamber (Super Xenon Weather Meter, SX75, Suga Test Instruments).

## Supplementary Material



## Notes

Theoretical content includes random Poisson statistics, cluster probability, and photocurrent analytical calculations for the device; modeling results cover numerical electromagnetic simulations with oblique incidence, size dependence, and dimer interparticle spacing; characterization data feature AFM images, interparticle spacing distribution, and substrate diffuse reflectance; and device performance results include photovoltaic characteristics, accelerated aging tests, the photon budget and color coordinate analysis based on the surface density, and slot-die coating experiments demonstrating scalability (PDF)
