# Supplementary material for: Selective Scatterers Improve Efficiency and Color Neutrality of Semitransparent Photovoltaics
Source: ACS Photonics. 2025 Nov 6;12(11):6458–67. doi: 10.1021/acsphotonics.5c02011 (PMC12636075; doi:10.1021/acsphotonics.5c02011)
Supplement: Supplementary file 1 [file ph5c02011_si_001.pdf]

Supplementary Information

Selective Scatterers Improve Efficiency and Color Neutrality of Semi-Transparent Photovoltaics

Zheheng Song[1*], Xi Lu[1], Oanh Vu[3], Jialu Song[3], Hiroshi Sugimoto[3], Minoru Fujii[3], Lars Berglund[2] and Ilya Sychugov[1*]

[1]Department of Applied Physics, KTH Royal Institute of Technology, Stockholm 11419, Sweden

[2] Department of Fibre and Polymer Technology, KTH Royal Institute of Technology, Stockholm 10044, Sweden

[3] Department of Electrical and Electronic Engineering, Kobe University, 657-8501, Japan

*ilyas@kth.se, *zheheng@kth.se

Supplementary Note 1 Poisson statistics

According to Poisson statistics, the cumulative distribution function (CDF) for having up to k particles within this interaction radius is given by a regularized upper incomplete gamma function.

$$F(k, N\pi r^2) = Q(k+1, N\pi r^2) = \frac{1}{k!}\int_{N\pi r^2}^{\infty} x^k \exp(-x)\, dx$$

$$= \frac{1}{k!}\int_{N\pi r^2}^{\infty} x^{N\pi r^2} exp\,(-N\pi r^2) d(N\pi r^2)$$

This function provides a mathematical framework to describe the likelihood of different point concentrations within a specified area. Let's consider a circle centered on a test particle, with the radius r representing the interparticle distance. By differentiating the CDF with respect to r, we obtain the PDF that describes the likelihood of having a distance r to the $n-th$ neighbor. Here, the index n is equal to $k+1$, since for $k=0$ (indicating no particles within the circle), the distance r corresponds to the distance to the first neighbor. Taking the derivative and applying standard integral tables immediately yields this PDF.

$$p_n(r) = \frac{dQ(k+1, N\pi r^2)}{dr} = \frac{2\pi N \left(\frac{k\Gamma(k+1)\gamma(k+1,\pi N r^2)}{2\pi N\Gamma(k+2)} + \frac{\Gamma(k+1)\gamma(k+1,\pi N r^2)}{2\pi N\Gamma(k+2)}\right)}{k!}$$

The Gamma function $\Gamma(k+1)$ serves as a normalization factor, extending the factorial to account for non-integer values, which is essential for accurately describing the distribution of particles. The lower incomplete gamma function $\gamma(k+1,\pi N r^2)$ captures the cumulative probability up to a certain radius r.

Supplementary Note 2 Photocurrent analysis of SiNPs on LSC device

Selective scatterers on the backside of an LSC device influence performance through multiple mechanisms. As shown in Figure S10, first, backscattered light within the escape cone partially absorbs into fluorophores before exiting, extending the optical path and enhancing absorption (retroreflector effect, similar to tapetum lucidum in animal eyes). This effect is wavelength-dependent. Second, backscattered light outside the escape cone undergoes TIR, either being absorbed by fluorophores, reaching the LSC edges for solar cell harvesting, or escaping via the scatterer layer. Lastly, the scatterer layer introduces additional scattering losses for waveguided luminescence from fluorophores. While the first two effects enhance photocurrent, the latter reduces it. The following sections provide quantitative estimates of each contribution.

1. Contribution of backscattered light inside the escape cone

To evaluate the effect of selective backscattering on LSC performance, we first consider the fraction of light backscattered into the escape cone. The backscattering cross section for this process, $\sigma_{EC}(\lambda)\ [cm^2]$, multiplied by the surface density of scatterers $N\ [cm^{-2}]$, gives the fraction of incoming light redirected into the escape cone, $R_{EC}(\lambda)\ = \sigma_{EC} \cdot N.$ This fraction acts on the incident solar spectrum $S(\lambda)\ [W/m^2]$

, modified by a single pass through the LSC:

$$S^*(\lambda) = S(\lambda) \cdot 0.96 \cdot \big(1 - A_{LSC}(\lambda)\big)[W/m^2]$$

where a 4% loss from the top interface is accounted for. The backscattered spectrum from selective scatterers is partially absorbed within the active layer, following:

$$A_{EC}(\lambda) = R_{EC}(\lambda) \cdot S^*(\lambda) \cdot A_{LSC}(\lambda)\ [W/m^2]$$

Integrating this over the spectrum and normalizing to the total incident solar power (1000 W/m^2) yields the fraction of solar light absorbed from escape cone photons, γ_{EC}.

2. Contribution of backscattered light outside the escape cone

For the backscattered light outside the escape cone (>42°), photons traveling at an average angle of ~60° relative to the normal undergo multiple transits through the active layer before reaching the same scattering interface. The optical path length for

such photons is approximately: $\frac{2d}{\cos(60°)} \approx 4d$, where d is the thickness of the active layer. The absorption fraction for these photons can be determined similarly: $\sigma_{WG}(\lambda) = \sigma_{BS}(\lambda) - \sigma_{EC}(\lambda)\ [cm^2]$ and $R_{WG}(\lambda) = \sigma_{WG} \cdot N$. The absorbed fraction for such four-pass photons is then:

$$A_{WG1}(\lambda) = R_{WG}(\lambda) \cdot S^*(\lambda) \cdot \left(1 - \left(1 - A_{LSC}(\lambda)\right)^4\right)\ [W/m^2]$$

Integrating over the spectrum and normalizing to the total solar power gives the fraction of solar light absorbed via these waveguided photons, A_{WG1}. Further absorption of waveguided photons can be analyzed through subsequent scattering events. After one additional bounce, the absorption loss coefficient for waveguiding at 60° incidence from the substrate side is given by:

$$L_{WG_{60}}(\lambda) = (\sigma_{FS_{60}}(\lambda) + \sigma_{ABS_{60}}(\lambda) + \sigma_{EC_{60}}(\lambda)) \cdot N$$

$$A_{WG2}(\lambda) = R_{WG}(\lambda) \cdot S^*(\lambda) \cdot \left(1 - A_{LSC}(\lambda)\right)^4 \cdot (1 - L_{WG_{60}}(\lambda)) \cdot \left(1 - \left(1 - A_{LSC}(\lambda)\right)^4\right)$$

$$A_{WG2}(\lambda) = A_{WG1}(\lambda) \cdot (1 - L_{WG_{60}}(\lambda)) \cdot \left(1 - A_{LSC}(\lambda)\right)^4 [W/m^2]$$

Repeating this process allows us to track the progressive absorption of waveguided photons over multiple bounces. Integrating over the solar spectrum gives the fraction A_{WG2}. The total contribution of absorbed photons to the LSC photocurrent can now be assessed. The photocurrent I_0 is proportional to the absorbed solar fraction γ, and the waveguiding efficiency[1,2]

$$I_0 \sim A_0 \cdot \eta_{WG}$$

With selective scatterers incorporated, the expression is modified as:

$$I_{ss} \sim (A_0 + A_{EC} + A_{WG1} + A_{WG2}) \cdot \eta_{WG_{ss}}$$

3.The loss caused by SiNPs

The loss coefficient of a single scattering event can be defined for a 60° from normal propagating waveguiding mode (average emission angle to the total internal reflection mode for isotopically luminescent QDs in a polymer slab) as $L_{WG_{60}}$taken at the luminescence intensity peak wavelength of $\lambda_0 = 850$ nm. Then the total waveguiding efficiency for the device will be reduced compared to the initial case as:

$$\eta_{WG_{ss}} = \eta_{WG} \cdot (1 - L_{WG_{60}})^{<N>}$$

where $< N >$ is an average number of bounces from the surface with SiNPs, which depends on the size of the device. The average number of bounces can be estimated considering isotropic emitters uniformly distributed in a thin polymer slab of thickness Δ. If the distance from the edge is x and the emission angle from horizontal plane is θ, the condition for luminescence to the waveguiding mode is $0 < \theta < \theta_{cr}$ with critical angle $\theta_{cr} \approx 48^o = \pi/3.75$. The average optical path in-plane x_0 for an LSC is known as $x_0 \approx a/2$ for a square LSC with side length a. The number of bounces is related to the angle θ and initial emission direction (towards or against scattering surface):

$$N_{\uparrow} = \frac{2x_0 tan\theta/\Delta + 3}{4}, N_{\downarrow} = \frac{2x_0 tan\theta/\Delta + 1}{4}$$

where the maximum number of bounces is (rounded down to the lower integer):

$$N_{max\uparrow} = \frac{2x_0 tan\theta_{cr}/\Delta + 3}{4}, N_{max\downarrow} = \frac{2x_0 tan\theta_{cr}/\Delta + 1}{4}$$

We need to find a probability density function for an isotropic emitter to produce photons experiencing N number of bounces from the scattering surface $\frac{dI}{dN}$. Isotropic emission implies a constant distribution of the luminescence intensity over the emitted angles:

$$\frac{dI}{d\theta} = \frac{dI}{dN} \cdot \frac{dN}{d\theta} = const$$

And the pdf becomes:

$$\frac{dI}{dN} = const \cdot \frac{d\theta}{dN}$$

Finding the derivative $\frac{d\theta}{dN}$ from the equations above and normalizing pdf $\frac{dI}{dN}$ so that its integral from zero to infinity is 1/2 for each direction we obtain:

$$\frac{dI}{dN_{\uparrow}} = \frac{1}{2\left(\text{atan}\left(\frac{3\Delta}{2\text{x}}\right) + \theta_{cr}\right)} \cdot \frac{2x_0\Delta}{4\left(N - \frac{3}{4}\right)^2 \Delta^2 + x_0{}^2}$$

$$\frac{dI}{dN_{\downarrow}} = \frac{1}{2\left(\text{atan}\left(\frac{\Delta}{2\text{x}}\right) + \theta_{cr}\right)} \cdot \frac{2x_0\Delta}{4\left(N - \frac{1}{4}\right)^2 \Delta^2 + x_0{}^2}$$

These are continuous functions, but since the number of bounces is an integer quantity it is more appropriate to operate with a probability mass function p_N for each direction:

$$p_{N\uparrow,\downarrow} = \int_{N-1}^{N} \frac{dI}{dN_{\uparrow,\downarrow}} dN$$

Then the average number of bounces can be calculated using the definition of an average:

$$< N >= \sum_{1}^{N_{max}} N \cdot (p_{N\uparrow} + p_{N\downarrow})$$

After numerical integration the average number of bounces from the scattering surface can be expressed with a convenient expression:

$$< N > \approx a/6$$

where a is a side length of a square LSC device in [cm]. If the backscattering reflection coefficient for the yellow part of the spectrum is $R_{BS} \approx 0.3$, which is needed to achieve color neutrality, and the quality factor of selective scatterers is $Q \approx 10$, the loss $L_{WG_{60}} = \frac{R_{BS}}{Q} = 0.03$. Then for a 20x20 cm^2 device $\eta_{WG\_SS} \approx 0.9\eta_{WG}$ and for a 30x30 cm^2 device $\eta_{WG\_SS} \approx 0.87\eta_{WG}$. In both cases photocurrent increase due to the absorption will dominate over the decrease due to increased scattering and a net increase of 5-10% of the photocurrent can still be expected.

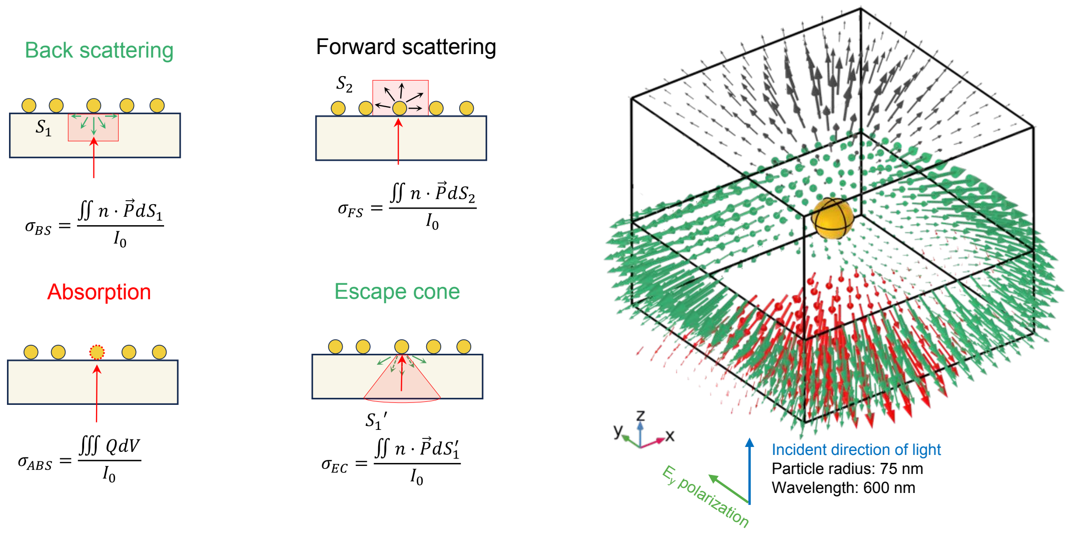

Figure S1. Definition and Extraction of Optical Cross Sections from Poynting Vector Analysis. Backscattering (BS), forward scattering (FS), absorption (ABS), and escape cone scattering (EC) cross sections are calculated by integrating the Poynting vector over selected surfaces or volumes, as illustrated in the top schematics. The escape cone is defined as the angular range within which scattered light can exit the system without undergoing total internal reflection. In this study, we determine the escape cone by analyzing the Poynting vector distribution from finite-element simulations. Specifically, we integrate the Poynting vector components over the surface while applying an angular condition: only vectors within the critical angle ($\theta_c < 42°$) contribute to the escape cone, while those exceeding this threshold ($\theta_c > 42°$) are associated with waveguiding modes. For the SiNP-on-glass system, the presence of the substrate modifies the escape conditions compared to a free-standing nanoparticle. Due to the refractive index contrast, light scattered at higher angles is more likely to be trapped within the substrate rather than escaping into free space. The provided figure visualizes these effects, with red arrows representing escaping Poynting vectors and blue arrows corresponding to waveguiding modes. The conditional integration approach ensures that only the relevant contributions to the escape cone are considered in the analysis.

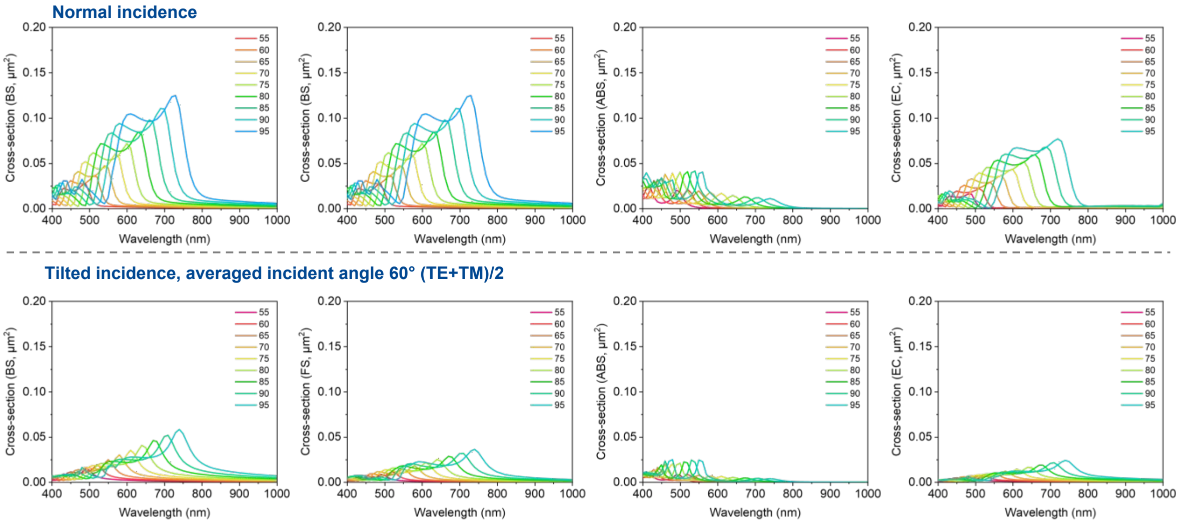

Figure S2. Size-Dependent Cross Sections under Normal and Oblique Incidence. Simulated spectral cross sections of BS, FS, ABS, and EC for SiNPs with radii ranging from 55 to 95 nm. The top row shows results under normal incidence, while the bottom row corresponds to a tilted (with 60° as averaged angle) incidence averaged over TE and TM polarization at 60°. All simulations are based on SiNPs-on-glass geometry.

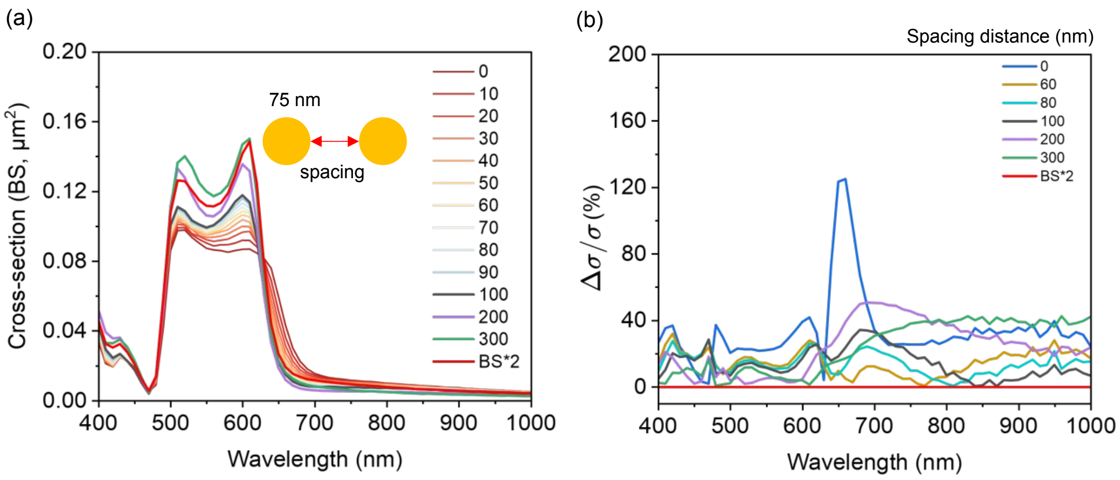

Figure S3. Coupling Effects on SiNP Dimers with Varying Interparticle Spacing. (a) Simulated BS spectra of SiNP dimers (r = 75 nm) with center-to-center spacing ranging from 0 to 300 nm. The red curve (BS×2) indicates the ideal individual case. (b) Relative variation in BS compared to the individual case, highlighting significant spectral deviations when spacing is <60 nm. A 20% threshold is used to define the onset of strong coupling.

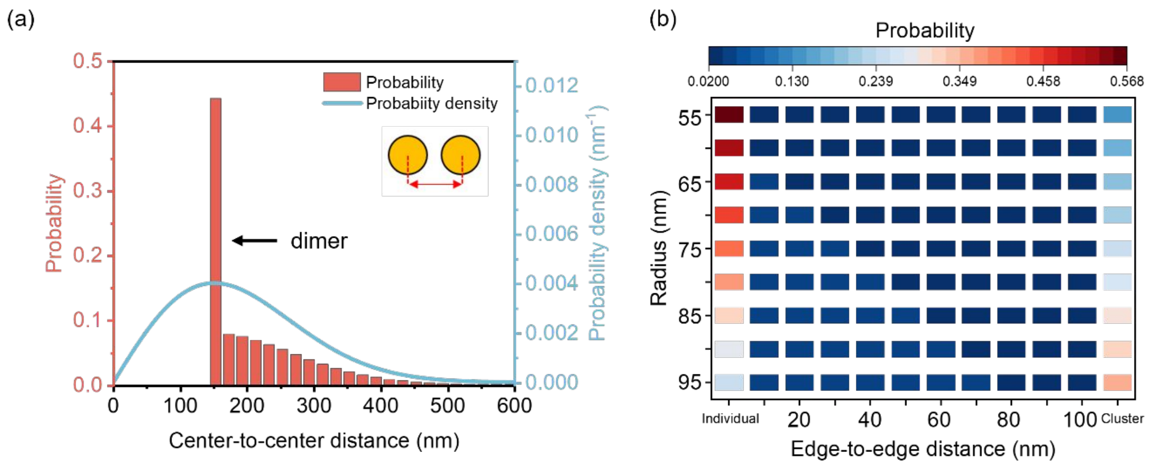

Figure S4. Statistical Probability of Dimer Formation Under Random Distribution. (a) Calculated probability and probability density function of SiNP center-to-center spacing under a Poisson process (r = 75 nm), showing ~44% probability of forming dimers (spacing <60 nm). (b) Heat map of dimer formation probability for various particle radii and edge-to-edge spacing distances, indicating increasing dimer probability with larger particle size.

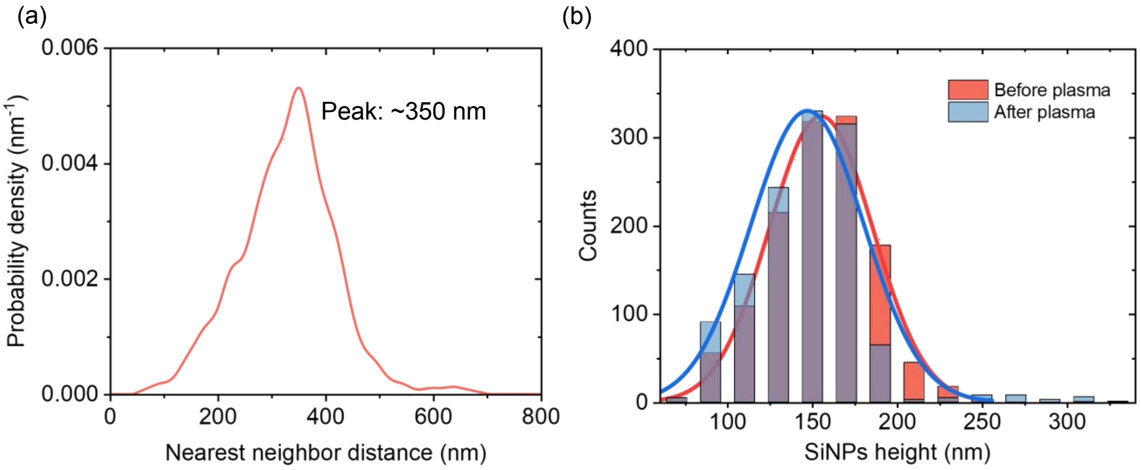

Figure S5. Statistical Analysis of Inter-Particle Spacing and Height Distribution. (a) Probability density distribution of nearest-neighbor distances for SiNPs (~5 NPs μm^{-2}) extracted from ImageJ and Python analysis, showing a peak around 350 nm, indicating spatially isolated particles. (b) SiNP height distribution before and after plasma treatment, showing shell removal and shrinkage.

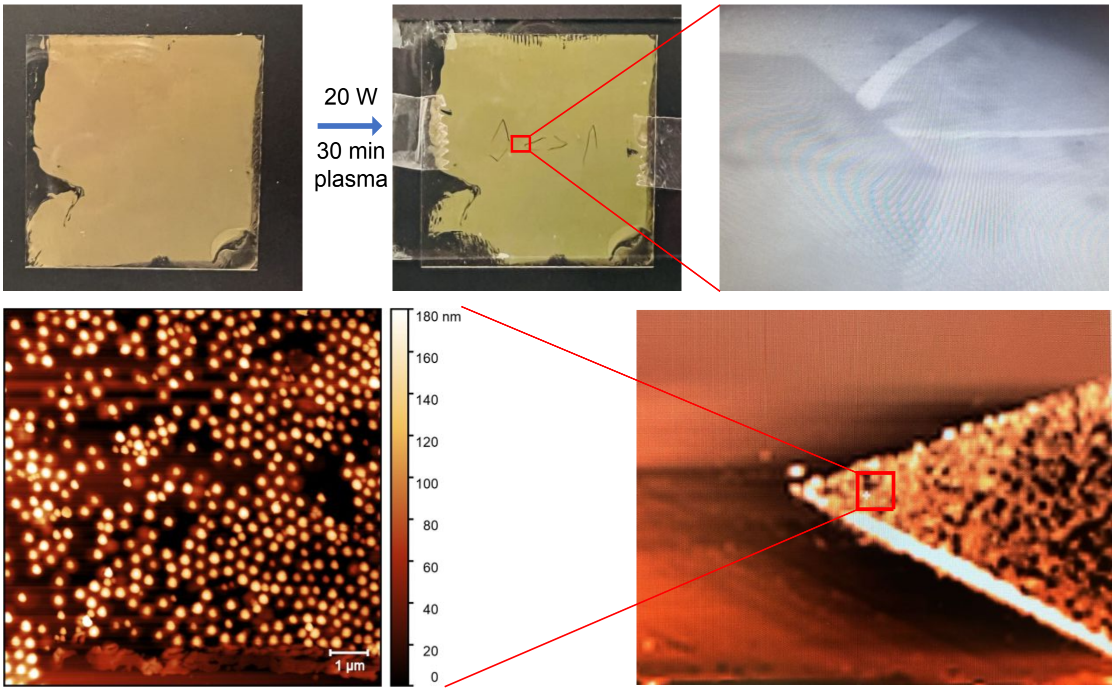

Figure S6. Experimental Strategy for Pre- and Post-Plasma Comparison on the Same Region. Photographs and AFM images showing the same SiNPs film region before and after 20 W, 30 min plasma treatment. Physical markers were applied using tweezers to enable spatial alignment. The same area was identified via CCD imaging and subsequently scanned using AFM to evaluate topographical changes.

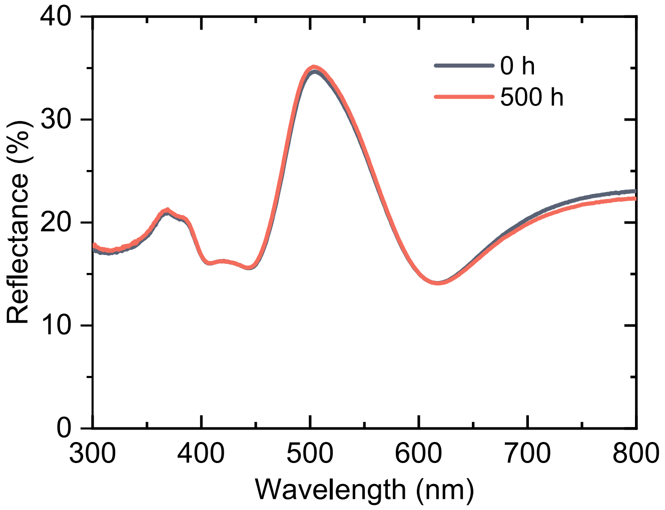

Figure S7. Accelerated Aging Test of Bare SiNPs. Reflectance measurements on a 100% monolayer film of bare SiNPs with 140 nm core diameter before and after 500 hr. Accelerated aging test in a UV climate chamber (Super Xenon Weather Meter, SX75, Suga Test Instruments). Irradiation level of 180 W/m^2 for given time corresponds to > 1-year of outdoor exposure. The slight change in reflectance is due to the difference in the measurement position (a few millimeters).

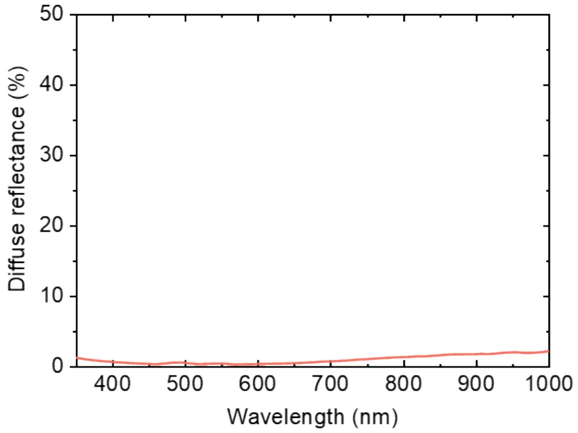

Figure S8. Diffuse Reflectance of the Bare Substrate.

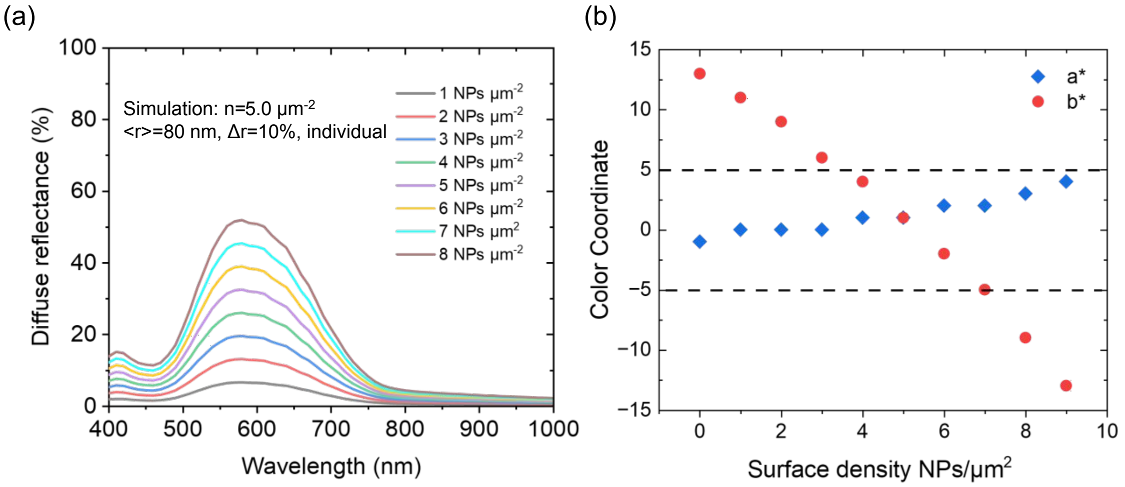

Figure S9. Simulated Scattering and Color Appearance for Different SiNP Surface Density. (a) Simulated diffuse reflectance spectra of SiNP films (r = 80 nm, Δr = 10%) with increasing surface densities (1–8 NPs μm^{-2}), assuming individual particles without clustering. (b) Extracted CIE a* and b* color coordinates as a function of surface density, indicating the evolution of visual color and neutrality window.

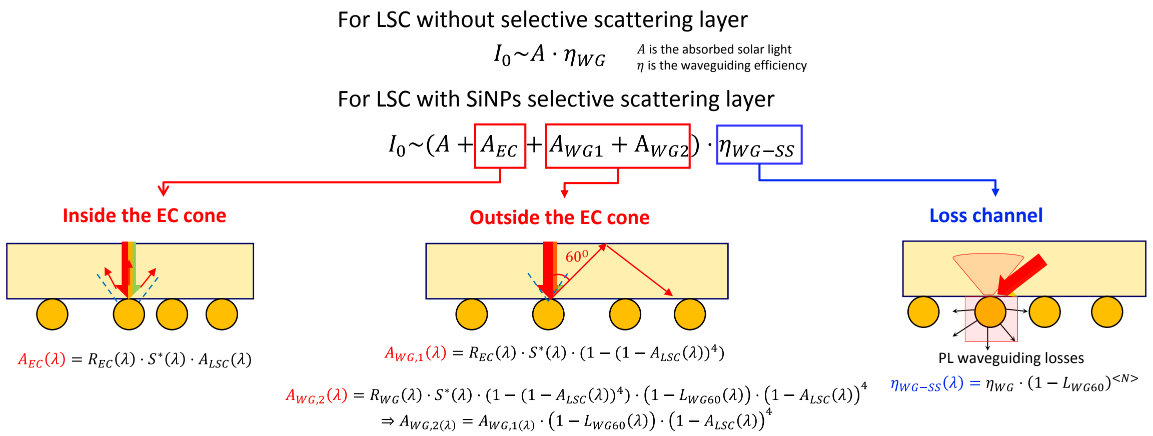

Figure S10. Schematic Model of SiNPs Scattering Contribution in LSC Systems. The presence of the SiNP selective scattering layer introduces three light interaction pathways in LSCs: (1) Inside the escape cone (EC): light is redirected into the substrate and absorbed by the LSC with probability A_{EC}. (2) Outside the EC: light is waveguided with absorption probabilities A_{WG1} and A_{WG2}, with the latter including additional losses. (3) Loss channel: parasitic PL waveguiding losses described by a reduced guiding efficiency η_{WG_SS}. These terms together redefine the effective light input I_0 in SiNP-integrated LSCs.

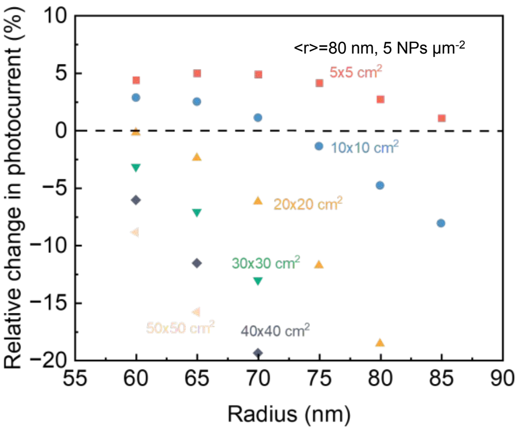

Figure S11. Impact of Dimer-Clustered SiNPs on LSC Photocurrent. Relative change in photocurrent for LSCs coated with clustered SiNPs (r ≥ 80 nm, surface density ~5 NPs μm^{-2}) across various device sizes.

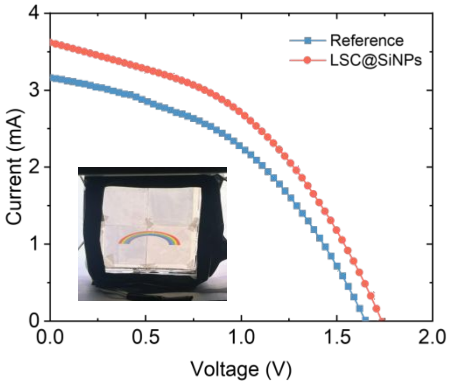

Figure S12. Photovoltaic I–V Characteristics of LSC Devices. Current–voltage curves of LSC@SiNPs and reference LSC devices under 1 sun (AM1.5G) illumination, corresponding to the results in Figure 5a. Fill factor improves slightly from 43.01% (reference) to 43.59% (LSC@SiNPs), while PCE increases from 0.072% to 0.100%. The inset shows the actual LSC@SiNPs device under test.

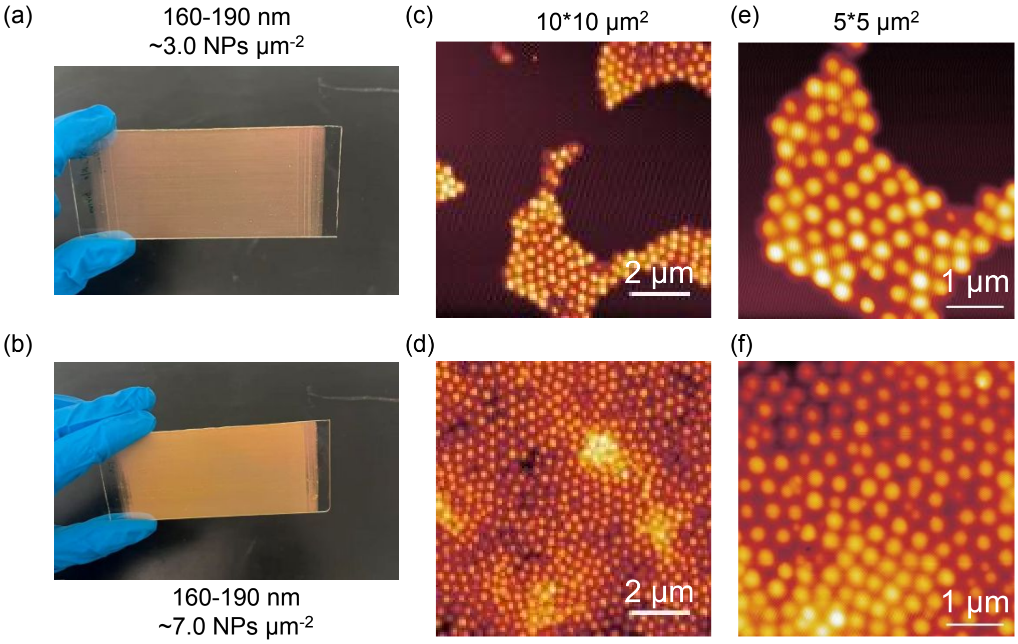

Figure S13. Scalability of Deposited SiNP Monolayers by Slot-die. Photographic images in (a) and (b) illustrate the large-area coating of SiNP monolayers achieved via multiple slot-die deposition passes, with nominal surface densities of ~3.0 NPs μm^{-2} and ~7.0 NPs μm^{-2}. The dark background is used to display the film's reflectance properties. The Atomic Force Microscopy (AFM) images in (c) and (e) correspond to the lower surface density (~3.0 NPs μm^{-2}) at 10*10 μm^2 and 5*5 μm^2 scales. Images (d) and (f) correspond to the higher surface density (~7.0 NPs μm^{-2}) at 10*10 μm^2 and 5*5 μm^2 scales. Slot-die coating successfully realizes large-area submonolayer deposition. The higher-density film appears macroscopically uniform across both 10*10 μm^2 and 5*5 μm^2 scales, indicating good control. For the lower-density film, while some minor clustering is visible in the 10*10 μm^2 image (c), individual nanoparticles are predominantly resolvable in the high-resolution 5*5 μm^2 image (e). Further optimization of slot-die fluid dynamics and solvent evaporation is required for precise surface density control, particularly for lower densities.

Reference

(1) Sychugov, I. Geometry Effects on Luminescence Solar Concentrator Efficiency: Analytical Treatment. *Appl. Opt.* **2020**, *59* (19), 5715. https://doi.org/10.1364/AO.393521.

(2) Sychugov, I. Analytical Description of a Luminescent Solar Concentrator. *Optica* **2019**, *6* (8), 1046. https://doi.org/10.1364/OPTICA.6.001046.
